# Invited Commentary: Less than Subtotal Parathyroidectomy for Multiple Endocrine Neoplasia Type 1 Primary Hyperparathyroidism: A Systematic Review and Meta-Analysis

**DOI:** 10.1007/s00268-022-06707-6

**Published:** 2022-08-30

**Authors:** F. Fausto Palazzo

**Affiliations:** Department of Endocrine & Thyroid Surgery, Hammersmith Hospital, London, England

Patients with an MEN1 genetic mutation nearly all present biochemical evidence of primary hyperparathyroidism (pHPT) in their teens. By definition these patients have unpredictably metachronous multiple gland parathyroid disease. Since nearly all pHPT patients are young there can be no doubt that a parathyroidectomy is indicated to counter the long-term deleterious effects of the excess parathyroid hormone on the bone, renal and cardiovascular systems [1]. However, the timing of surgery, in other words at what point in the disease natural history to operate, and what operation to perform remain somewhat less categorically agreed.

The issue of timing of the parathyroid surgery is debated because whilst early intervention limits the risk of end organ damage it tends to be associated with macroscopically more subtle disease and a longer life span for recurrence to occur. Earlier intervention also provides a longer length of patient life to live with the any adverse surgical outcome, in particular hypoparathyroidism. The accepted consensus appears to be that surgery should ideally happen before end organ damage is present, before the biochemistry becomes severe and before a pregnancy in female patients. Once the decision to perform a parathyroidectomy has been taken, usually a joint decision between the patient, the endocrinologist and the surgeon, the optimal surgical strategy in MEN1 patients has also to be agreed.

The surgical approach to MEN1 parathyroidectomy has changed over the past decades. A total parathyroidectomy with or without thymectomy and parathyroid autotransplantation in the brachioradialis muscle was popular in central Europe but appears to have lost favour because of the variable efficacy of parathyroid autotransplantation. It has been largely replaced by a subtotal parathyroidectomy with removal of 3 or 3.5 glands often with a cervical thymectomy [2]. The arrival of increasingly effective parathyroid localisation in sporadic pHPT has opened new possibilities, specifically that of a “less than subtotal or total parathyroidectomy” (LSTP) option.

This systematic review of the 2 currently most practiced parathyroidectomy strategies by Dr Bouriez and colleagues highlights the advantages and disadvantages of a LSTP and subtotal parathyroidectomy in MEN1 patients [3]. The LSTP usually consists in a unilateral operation where the pre-operatively identified presumed dominant parathyroid and the ipsilateral second parathyroid and thymus are removed. This is an encouraging proposition since it avoids re-exploration of a scarred neck at the almost inevitable subsequent re-operation [4]. However, patient selection is essential to avoid what many consider an unacceptably high—one quarter of patients—persistence rate and an earlier recurrence rate. However the exponents of LSTP will counter-argue that a subtotal parathyroidectomy, especially in the 3.5 gland parathyroidectomy guise comes with an excessively high permanent hypoparathyroidism rate.

The advantages of a unilateral approach in MEN1 parathyroidectomy can only be obtained if the truly unilateral dominant disease can be reliably identified. The parathyroid dominance can be functional—sestamibi or Choline PET—or based on size using US or CT. However sestamibi scans tend to be negative in multiple gland disease so does the presence of a positive alternative imaging modality make a LSTP wise?

The 2 approaches discussed in this manuscript are not mutually exclusive and it seems clear that an approach tailored to each patient circumstance is appropriate [5]. Minimising the negative aspect of each surgical strategy is a desirable goal. A lower morbidity—hypoparathyroidism in particular—in a subtotal parathyroidectomy is required. Meanwhile in the LSTP approach the need to identify a localisation modality that best predicts which patients can have LSTP with minimal persistence and early recurrence without excessive cost and radiation exposure is yet to be identified.
